# Whole-Genome Scans Provide Evidence of Adaptive Evolution in Malawian *Plasmodium falciparum* Isolates

**DOI:** 10.1093/infdis/jiu349

**Published:** 2014-06-19

**Authors:** Harold Ocholla, Mark D. Preston, Mwapatsa Mipando, Anja T. R. Jensen, Susana Campino, Bronwyn MacInnis, Daniel Alcock, Anja Terlouw, Issaka Zongo, Jean-Bosco Oudraogo, Abdoulaye A. Djimde, Samuel Assefa, Ogobara K. Doumbo, Steffen Borrmann, Alexis Nzila, Kevin Marsh, Rick M. Fairhurst, Francois Nosten, Tim J. C. Anderson, Dominic P. Kwiatkowski, Alister Craig, Taane G. Clark, Jacqui Montgomery

**Affiliations:** 1 Malawi-Liverpool-Wellcome Trust Clinical Research Programme; 2 Department of Physiology, College of Medicine, University of Malawi, Blantyre; 3 Liverpool School of Tropical Medicine, Pembroke Place, Liverpool; 4 Faculty of Infectious and Tropical Diseases, London School of Hygiene and Tropical Medicine; 5 Wellcome Trust Sanger Institute, Hinxton; 6 Wellcome Trust Centre for Human Genetics, University of Oxford, United Kingdom; 7 Centre for Medical Parasitology, Department of International Health, Immunology and Microbiology, University of Copenhagen; 8 Department of Infectious Diseases, Copenhagen University Hospital, Denmark; 9 Institut de Recherche en Sciences de la Sant, Bobo-Dioulasso, Burkina Faso; 10 Malaria Research and Training Centre, Faculty of Medicine, Pharmacy and Dentistry, University of Bamako, Mali; 11 Institute of Tropical Medicine, University of Tübingen, Germany; 12 Department of Biology, King Fahd University of Petroleum and Minerals, Dhahran, Saudi Arabia; 13 KEMRI–Wellcome Trust Research Programme, Kilifi, Kenya; 14 Laboratory of Malaria and Vector Research, National Institute of Allergy and Infectious Diseases, National Institutes of Health, Bethesda, Maryland; 15 Texas Biomedical Research Institute, San Antonio, Texas; 16 Centre for Tropical Medicine, Nuffield Department of Medicine, University of Oxford, United Kingdom; 17 Shoklo Malaria Research Unit, Mahidol-Oxford Tropical Medicine Research Unit, Faculty of Tropical Medicine, Mahidol University, Mae Sot, Thailand

**Keywords:** *Plasmodium falciparum*, genomes, genetic epidemiology, Malawi

## Abstract

**Background:**

Selection by host immunity and antimalarial drugs has driven extensive adaptive evolution in *Plasmodium falciparum* and continues to produce ever-changing landscapes of genetic variation.

**Methods:**

We performed whole-genome sequencing of 69 *P. falciparum* isolates from Malawi and used population genetics approaches to investigate genetic diversity and population structure and identify loci under selection.

**Results:**

High genetic diversity (π = 2.4 × 10^−4^), moderately high multiplicity of infection (2.7), and low linkage disequilibrium (500-bp) were observed in Chikhwawa District, Malawi, an area of high malaria transmission. Allele frequency–based tests provided evidence of recent population growth in Malawi and detected potential targets of host immunity and candidate vaccine antigens. Comparison of the sequence variation between isolates from Malawi and those from 5 geographically dispersed countries (Kenya, Burkina Faso, Mali, Cambodia, and Thailand) detected population genetic differences between Africa and Asia, within Southeast Asia, and within Africa. Haplotype-based tests of selection to sequence data from all 6 populations identified signals of directional selection at known drug-resistance loci, including *pfcrt*, *pfdhps*, *pfmdr1*, and *pfgch1*.

**Conclusions:**

The sequence variations observed at drug-resistance loci reflect differences in each country's historical use of antimalarial drugs and may be useful in formulating local malaria treatment guidelines.

An estimated 3.3 billion people worldwide are at risk of malaria. The majority of cases (81%) and deaths (91%) occur in sub-Saharan Africa, where children <5 years old and pregnant women bear the greatest burden of disease [1]. In Malawi, almost the entire population is at risk of developing *Plasmodium falciparum* malaria, which accounts for 40% of hospitalizations of children <5 years old, 34% of outpatient visits by children of all ages, and 40% of hospital deaths. Year-round malaria transmission occurs in almost every part of the country and peaks during the annual rainy season, from December to May [2]. Since 2005–2007, Malawi and its external donors have scaled up malaria control interventions; coverage for long-lasting insecticide-treated bed nets (LLINs) has reached 60%, intensive indoor residual spraying (IRS) has been launched and expanded in districts with high transmission rates, and artemisinin-based combination therapy (ACT) has replaced sulfadoxine-pyrimethamine (SP) as the first-line treatment for malaria [1, 3]. However, childhood cases of malaria have not declined since 2001 [3], and the overall prevalence of anemia and parasitemia have not reflected the scaled up access to malaria interventions. Recently, the prevalence of parasitemia in rural children was higher than that in urban children (47% vs 15%), and the prevalence in the central region of Malawi (50%) was higher than that in the southern (42%) and northern (23%) regions (Malawi National Malaria Indicator Survey, 2010, http://files.givewell.org/files/DWDA%202009/AMF/Malawi_MIS_2010_Final.pdf). On the other hand, scaled up malaria interventions (eg, LLIN use) have benefitted pregnant women by reducing the prevalence of peripheral parasitemia (from 23.5% to 5.0%) and placental malaria (from 25.2% to 6.8%) [4].

An integrated approach to malaria control is essential, and the application of genomics is one area that can provide biological insights into important evolutionary forces in *P. falciparum.* An improved understanding of the parasite's genetic diversity, population structure, and natural selection will have practical implications for developing methods of disease control. In particular, genetic variation enables the parasite to overcome host immunity, antimalarial drugs, and vaccines to establish persistent infections and increase transmission [5–7]. In this study, we analyzed whole-genome sequence variation in 69 *P. falciparum* isolates obtained from children in the Chikhwawa district of Malawi to investigate the genomic epidemiology of *P. falciparum* in this area and to explore the impact of host immunity and antimalarial drugs on the parasite population. We have used allele frequency–based approaches to infer the parasite's demographic history in Malawi and discover genetic loci likely to be under balancing selection [8]. Results from our analysis of genetic diversity, linkage disequilibrium (LD), and multiplicity of infection (MOI) are consistent with the high level of malaria transmission in Chikhwawa District. Comparison of sequence variation in parasite populations from Malawi and Kenya, Burkina Faso, Mali, Cambodia, and Thailand, using haplotype-based tests and population differentiation metrics (*F_ST_*), identified signals of directional selection at known drug-resistance loci, including *pfcrt*, *pfdhps*, *pfmdr1*, and *pfgch1*. These findings highlight potential roles of genomics in guiding malaria control efforts, such as monitoring of key drug biomarkers and informing drug policy, and changes in parasite populations structure that correspond to and can predict changes in malaria epidemiology.

## METHODS

### Study Site and Patients

The study was performed in southern Malawi's Chikhwawa District (16°1′ S, 34°47′ E), a rural area of intense perennial malaria transmission (entomological inoculation rate, 183 infective bites/person/year) [9]. It is approximately 70 m above sea level, divided throughout its length by the Shire River, and prone to flooding during the wet season. It has a tropical climate with a mean annual temperature of 26°C, a single wet season from December to May, and an annual rainfall level of approximately 770 mm [9]. With an annual average infection prevalence in 2–10-year-old children that exceeds 40% [10], Chikhwawa District has one of the highest malaria transmission rates and is 1 of 12 sites in Malawi chosen for intensive antimalarial interventions: IRS, extensive LLIN coverage, and ACT. SP is used for intermittent preventive treatment in pregnancy (IPTp) [11].

Permission to conduct the study was granted by ethics committees of the College of Medicine, Malawi and the Liverpool School of Tropical Medicine. Written informed consent was obtained from a parent or guardian of each child.

### Sample Collection and Processing

Between December 2010 and July 2011, 93 whole-blood samples were collected from children participating in a clinical trial at Chikhwawa District Hospital. Blood was depleted of leukocytes by CF11 column filtration [12], and genomic DNA was extracted using the QIAamp DNA Blood Midi Kit (Qiagen). Human and *P. falciparum* DNA levels were quantified using PicoGreen analysis and quantitative real-time polymerase chain reaction (PCR), using the Applied Biosystems stepOne RT-PCR system [13]. Samples used for sequencing yielded >50 ng of DNA and had <80% human DNA contamination. Samples were sequenced by the Illumina Genome Analyzer IIx or the Illumina HiSeq 2000, using the manufacturer's recommended protocol [14], with a minimum of 76-bp paired-end reads.

### Data Processing: Alignment, Single-Nucleotide Polymorphism (SNP) Discovery, and Quality Filtering

Detailed description of the analysis pipeline has been described elsewhere [15, 16]. Briefly, short reads for all 93 samples were mapped to the 3D7 reference genome (version 3.0), using SMALT (http://www.sanger.ac.uk/resources/software/smalt) with default parameters, and SNPs were called using SAMtools (http://samtools.sourceforge.net). This process identified 115 965 SNPs across the 93 samples, 24 of which were discarded because of very low coverage (average coverage across the whole genome, <10-fold). For the remaining 69 samples (average coverage across whole genome, >35-fold), we retained 88 655 high-quality SNPs (76.4%) in their nuclear genomes that met the following criteria: (1) biallelic; (2) quality scores of >30 (error rate, <1 per 1000-bp); (3) not in genomic positions at the very extremes of the coverage distribution (sample average coverage, <10-fold or >2000-fold), which could reflect deletions or copy number variants, respectively [17]; (4) not located in subtelomeric regions, the hypervariable *var*, *rifin*, and *stevor* gene families, or regions of low uniqueness; and (5) no SNP positions with ≥3 problematic genotypes (missing or mixed). Uniqueness was calculated by a sliding 54-bp window of contiguous sequence across the 3D7 reference genome and detecting the presence of this motif elsewhere in the genome. Only SNPs that were in unique positions were retained. Genotypes at SNP positions were called using ratios of coverage. Heterozygous calls were converted to the majority genotype if the coverage ratio was 80:20 or greater [15, 18], and the resulting majority allele data were used for further analysis. The filtering of samples and SNPs with mixed genotypes minimized any potential effects of multiplicity of infection. Progeny of the HB3 × DD2 cross (n = 25; 35 832 SNPs [19]) and other population data (Kenya, n = 37; Burkina Faso, n = 40; Mali, n = 40; Cambodia, n = 80; Thailand, n = 80; 294 187 SNPs; [16]) were processed in the same way. The 4 mitochondrial SNPs (*mt772, mt1692, mt4179*, and *mt4952*) were extracted from the alignments and the genotypes called as described above. Public accession numbers for sequence data are contained in SRA studies (ERP000190 and ERP000199; http://www.malariagen.net).

### Population Genetics

For the high-quality SNPs (n = 88 655) in the Malawian population, we estimated the genetic diversity by calculating the average pair-wise nucleotide diversity (π). We used *DnaSP* [20] software to compute the allele frequency-based Tajima *D* test [8] and Fu and Li's *D* and *F* metrics [21] to identify genes under balancing selection. Results from Fu and Li's *D* and *F* metrics correlated highly with those from the Tajima *D* test (Spearman ρ, 0.85) and were not analyzed further. To detect signals of directional selection, the integrated haplotype score (iHS) [22] was used, and its *P* values were computed from standardized values based on a 2-tailed conversion from a Gaussian distribution [23]. The MOI in Malawi was estimated using a novel method of counting the unique haplotypes formed by polymorphism on paired sequencing reads [24]. We estimated recombination rate in this population using 2 progeny crosses. First, rates from the 7G8 × GB4 (Ghana × Brazil origin) were accessed [25]. Second, rates from the HB3 × DD2 (Honduras × Indo-China origin) cross [19] were estimated using the R QTL library (http://www.rqtl.org). For comparisons between populations (Malawi vs others, n = 294 187 SNPs), we first applied the principal component analysis (PCA), based on a matrix of pair-wise identity by state values, followed by the cross-population long-range LD method, XP-EHH [26] and population differentiation metric *F_ST_* [27]. *P* values for the XP-EHH estimates were calculated using a Gaussian approximation. A significance threshold of *P* < .0006 was established for both iHS and XP-EHH, using a simulation approach. We used the ranked *F_ST_* statistics to identify the informative polymorphism driving the clustering in the PCA. LD was measured using the *r^2^* metric [28], calculated for pairs of SNPs with different physical separation up to 10-kb, using a sliding window approach. The R statistical package was used to analyze the results.

## RESULTS AND DISCUSSION

### Genetic Diversity and Signatures of Selection Within the Malawian Population

We identified 88 655 high-quality biallelic SNPs (approximately 1 SNP per 260 bp, compared with approximately 1 per 266 bp among isolates worldwide [18]) in robust genomic regions of the 69 samples with good sequence coverage. The overall π in Malawian samples (0.00025; 95% confidence interval, .00023–.00026) is consistent with estimates of high genetic diversity observed in African samples, especially in regions of high transmission [5, 23, 29]. We observed high variability in π across chromosomes (minimum, 0.00011, chromosome 3; maximum, 0.00037, chromosome 4), probably because of variation in recombination rates. Analysis of antigenic regions of extremely high diversity (200 genes; average π, 0.00050) led to a minor decrease in diversity (average π, 0.00023; *P* = .035, by the Wilcoxon signed rank test on differences). Regions with elevated diversity may encode an antigenic or polymorphic locus that may be useful for vaccine approaches. Modest positive correlations were observed between recombination rate and diversity (Spearman ρ, r = 0.075 for 7G8 × GB4; r = 0.101 for HB3 × DD2).

**Figure 1.**
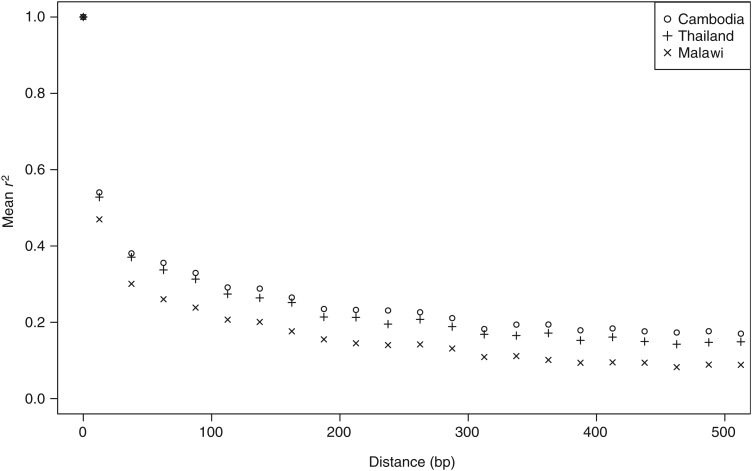
Decay in linkage disequilibrium (LD) between populations. LD decays rapidly in Malawi, compared with Southeast Asian populations.

To examine the potential effects of recombination, we looked at levels of LD in the Malawian population, which decayed rapidly within a few 100 bp and reached a baseline level within 500 bp. The decay in LD was more rapid in Malawian parasites than in Asian parasites, as previously reported (MAF, 10%; Figure 1) [16, 18]. The lower LD in Malawian samples suggests high levels of outcrossing (and effective recombination), as expected in areas of relatively high transmission intensity [23]. Indeed, a high EIR (183 infective bites/person/year) has previously been described for this Malawian population [9], consistent with a previous report of high estimated MOI (median, 2.7; range, 1–10) [30], and was higher than 2 Southeast Asian populations (medians, <1.5) [24]. In this latter study, <10% of samples had multiple infections, compared with approximately 50% of African samples, also reflecting differing transmission patterns [24]. Further examination of LD identified 40 genomic regions (containing at least 5 SNPs) with high average *r^2^* values (>0.5) that extended beyond 1 kb (Supplementary Table 1). We found no evidence of low recombination rates in these regions, and none of the genes were in the lowest 10th percentile of the recombination rates distribution for either genetic cross. One particular gene, *piesp2* (*PF3D7_0501200*, which encodes a parasite-infected erythrocyte surface protein), showed a pattern of LD extending to 1 kb, suggesting that this region is under recent positive selection.

**Figure 2.**
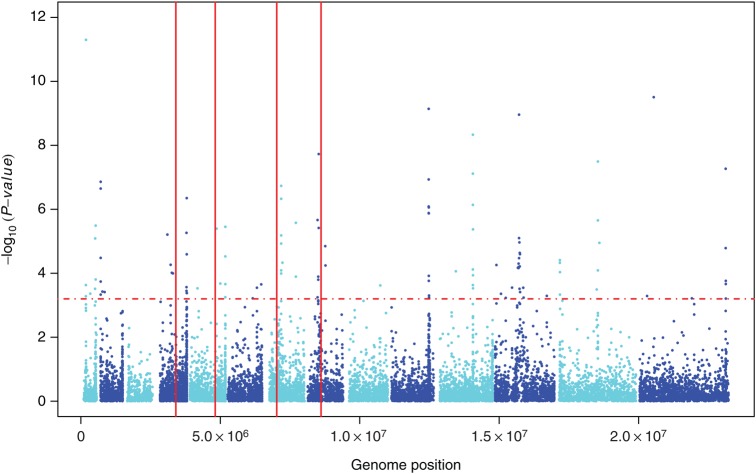
Positive directional selection in the Malawian *Plasmodium falciparum* population. Dashed line indicates genome-wide integrated haplotype scores at a significance threshold of *P* < .0006. Vertical lines indicate (from left) locations of *pfdhfr, pfmdr1*, *pfcrt*, and *pfdhps*, respectively.

Computation of iHS for the Malawian SNPs identified 14 chromosomal loci likely to be under positive directional selection (*P* < .0006; Table 1 and Figure 2). These include regions surrounding 2 SP resistance loci (*pfdhps* and *pfgch1*) that extend to several genes, suggesting that drug selection has produced chromosomal segments of selective sweeps. We did not detect selection signals at *pfcrt*, but there was evidence of selection within 3 kb of the gene. This may be expected with regression back to the *pfcrt* wild-type alleles after withdrawal of chloroquine (CQ) from Malawi >15 years ago. Signals were also detected within 10 kb of *pfubp1,* a homologue of a *Plasmodium chabaudi* gene linked to artemisinin resistance. In Kenya, *pfubp1* alleles were recently found to be under directional selection and associated with reduced in vitro susceptibility to artemisinin [31]. Positive directional selection signals were also evident in *msp6* and *msp3.8*, *pfama1*, *trap*, and *msp7* on chromosomes 10, 11, 13, and 14, respectively. These genes are expressed predominantly in merozoites and are thought to be primarily under balancing selection, consistent with other observations in Asian and African populations [31–33]. These non–drug-related drivers of directional selection, as well as antigenic loci that modulate drug resistance (eg, *msp3.8* and members of the *clag* gene family [34, 35]), require further investigation. As expected, Malawian samples have the African-specific K189T mutation in *PF3D7_1343700* but none of the so-called K13-propeller mutations (eg, C580Y, R539T, or Y493H) associated with artemisinin resistance in Cambodia [36].

**Table 1.** Genetic Loci Under Recent Positive Directional Selection in Malawi, Identified Using the Integrated Haplotype Score at A Significance Threshold of *P* < .0006

Chromosome	Start	Stop	Locus Characteristic(s)
1	178 726	180 317	Within approximately 10 kb of *pfubp1*
	512 851	558 256	Contains 7 genes (*PF3D7_0113700–PF3D7_0114500*)
2	842 699	855 734	Contains 3 genes, including *clag2*
4	1 065 176	1 144 415	Contains 16 genes (*PF3D7_0423600–PF3D7_0425400*)
5	966 314	1 181 373	Approximately 4-kb from *pfmdr1*
7	409 122	470 642	Approximately 3-kb from *pfcrt*
	507 357	665 385	Contains 27 genes (*PF3D7_0711700–PF3D7_0714500*)
	1 358 889	1 380 385	Contains 4 genes, including *eba175*
8	449 188	585 854	Contains 28 genes, including *pfdhps*
10	1 389 354	1 434 268	Contains 12 genes, including *msp3, 3.3, 3.8, 6,* and *11*
11	1 294 082	1 295 369	*pfama1*
12	800 894	1 059 078	Contains 58 genes, including *pfgch1*
13	102 848	106 661	*trap*
14	2 982 003	3 149 504	Contains 32 genes

Interrogation of the allele frequency spectrum of different classes of nucleotide sites showed an excess of rare alleles (Supplementary Figure 1), with coding, nonsynonymous, synonymous, and intergenic sites more skewed than expected under neutrality. This observation indicates recent population expansion, as has been demonstrated in other African populations [23, 37]. Use of the allele frequency-based Tajima *D* test to evaluate each polymorphic gene across the genome identified regions likely to be under balancing selection. There was a strong positive correlation between *π* and Tajima *D* values (Spearman *ρ*, 0.532), providing potential evidence of increasing diversity with balancing selection. For the 2073 genes (51.1%) with at least 5 SNPs computed for Tajima *D*, the majority of values (96.5%) were negative. To negate any confounding effects of population expansion and difficulties in establishing significance levels, we report 19 genes with Tajima *D* values of >1 (Table 2). These include a significant overrepresentation of genes (*msp3.8, msp3, dbl-msp, eba-175, ama1,* and *surfin4.2*) involved in merozoite invasion of erythrocytes and previously reported to be under immune selection [31, 37, 38]. The identification of additional genes potentially under immune selection may suggest additional proteins as candidate vaccine antigens.

**Table 2.** Genes Under Balancing Selection, Defined as ≥5 Single-Nucleotide Polymorphisms and a Tajima D of ≥ 1

Gene	Tajima *D*	Start	Stop	Protein Encoded by Gene
PF3D7_1036300	3.26	1 432 702	1 434 553	Merozoite surface protein 3.8
PF3D7_0710000	2.52	447 902	457 801	Conserved *Plasmodium* protein; unknown function
PF3D7_0425400	2.06	1 144 011	1 144 822	*Plasmodium* exported protein (PHISTa); unknown function
PF3D7_0221000	1.98	848 124	849 107	*Plasmodium* exported protein; unknown function
PF3D7_0709300	1.96	414 302	421 420	Cg2 protein
PF3D7_0710200	1.94	463 705	471 598	Conserved *Plasmodium* protein; unknown function
PF3D7_0424400	1.85	1 100 085	1 102 381	Surface-associated interspersed protein 4.2 (SURFIN 4.2)
PF3D7_0630800	1.83	1 288 574	1 290 718	Conserved *Plasmodium* protein; unknown function
PF3D7_1133400	1.72	1 293 957	1 295 622	Apical membrane antigen 1
PF3D7_1149600	1.59	2 001 079	2 003 312	DnaJ protein; putative
PF3D7_1126100	1.57	1 018 557	1 021 025	Autophagy-related protein 7; putative
PF3D7_0731500	1.56	1 358 502	1 362 925	Erythrocyte binding antigen-175
PF3D7_0104100	1.49	178 094	180 554	Conserved *Plasmodium* membrane protein; unknown function
PF3D7_0516300	1.39	679 096	680 745	Transfer RNA pseudouridine synthase; putative
PF3D7_1035400	1.37	1 404 453	1 405 160	Merozoite surface protein 3
PF3D7_0113800	1.24	527 113	536 327	Duffy binding-like–containing protein; unknown function
PF3D7_0630300	1.15	1 260 750	1 269 383	DNA polymerase epsilon, catalytic subunit a; putative
PF3D7_0103600	1.03	161 480	165 521	Adenosine triphosphate–dependent RNA helicase; putative
PF3D7_1035700	1.01	1 413 250	1 415 182	Duffy binding-like merozoite surface protein

### Comparison of the Malawian Population to Other Populations

Of the 294 187 high-quality SNPs identified across the 6 populations (Malawi, 46% of SNP sites observed; Kenya, 30%; Burkina Faso, 33%; Mali, 37%; Cambodia, 22%; and Thailand, 23%), only 8% were polymorphic in Malawi. The comparison of Malawian samples to samples from each of the 5 populations, using the cross population long-range haplotype method (XP-EHH), identified regions potentially under positive directional selection at or near known drug-resistance loci (*pfdhps*, *pfcrt*, and *pfgch1*; Table 3). Although low recombination rates may confound the directional selection interpretation, a follow-up analysis of genetic cross-progeny showed that these identified regions do not have low recombination rates.

**Table 3.** Genetic Loci Under Directional Selection in All 6 Populations Identified Using XP-EHH at a Significance Threshold of *P* < .0006

Population, Chromosome	Start	End	Locus Characteristic(s)
Malawi			
1	180 314	193 846	Contains 3 genes, including *pfubp1*
	487 895	489 460	Contains gene encoding glutamic acid–rich protein
4	755 433	881 703	Approximately 6 kb from *pfdhfr*
	990 908	991 327	Contains gene encoding conserved *Plasmodium* protein; unknown function
5	1 042 259	1 109 432	Approximately 8 kb from *pfmdr1*
8	532 499	585 854	Contains 14 genes, including *pfdhps*
10	1 326 109	1 327 397	Contains gene encoding S-adenosylmethionine decarboxylase/ornithine decarboxylase
12	461 137	473 836	Contains 5 genes (*PF3D7_1210200–PF3D7_1210600*)
	946 416	954 490	Approximately 30 kb from *pfgch1*
	983 440	1 016 281	Contains 9 genes (*PF3D7_1224200*–*PF3D7_1225000*)
	1 004 000	1 022 661	Contains 5 genes (*PF3D7_1224700–PF3D7_1225100*)
13	1 465 713	1 465 965	Contains gene encoding sporozoite surface protein 2 (*trap*)
14	1 688 102	1 688 881	Contains gene encoding serine/threonine protein kinase; putative
	2 135 779	2 137 007	Contains rhoptry neck protein 2 (*ron2*)
Kenya			
6	1 116 365	1 222 963	Contains 20 genes (*PF3D7_0627800–PF3D7_0629700*)
7	376 423	417 661	*pfcrt*
8	467 328	468 623	Contains gene encoding asparagine-rich antigen Pfa55-14
Mali			
6	1 205 649	1 290 486	Contains 16 genes (*PF3D7_0629300–PF3D7_0630800*)
7	376 423	470 941	*pfcrt*
	505 661	614 698	Contains 13 genes (*PF3D7_0711500*–PF3D7_0713500)
	1 100 440	1 326 844	Contains 48 genes (*PF3D7_0726200*–*PF3D7_0731900*)
8	468 447	469 357	Contains gene encoding asparagine-rich antigen Pfa55-14
Burkina Faso			
1	487 895	489 267	Contains gene encoding glutamic acid–rich protein
7	432 780	507 284	Within 26 kb of *pfcrt*
	908 940	918 733	Contains 3 genes (*PF3D7_0721000–PF3D7_0721200*)
8	416 971	422 505	Contains gene encoding plasmepsin X
Thailand			
4	709 512	771 505	*pfdhfr*
7	339 092	451 640	*pfcrt*
8	468 447	586 054	*pfdhps*
	703 454	712 742	Contains 5 genes (*PF3D7_0814600–PF3D7_0815100*)
Cambodia			
6	1 109 423	1 135 810	Contains 5 genes (*PF3D7_0627700–PF3D7_0628100*)
7	332 719	453 986	*pfcrt*
	875 300	931 176	Contains 17 genes (*PF3D7_0720000*–*PF3D7_0721500*)
8	468 669	479 732	Contains gene encoding asparagine-rich antigen Pfa55-14

In Malawi, directional selection at *pfdhps* is probably due to high SP pressure, while *pfcrt* selection in Burkina Faso, Mali, Cambodia, and Thailand is likely due to high CQ pressure. Signals detected at *pfcrt* and *pfdhps* in Kenya and Thailand are also indicative of CQ and SP selection, respectively. The lack of evidence for selection at *pfcrt* in Malawi reflects the withdrawal of CQ and subsequent increase in the ancestral CQ-susceptible allele frequency, due to the re-expansion of a persistent minority population of CQ-susceptible parasites. This observation suggests that parasites carrying ancestral *pfcrt* alleles have greater fitness in the absence of CQ pressure [39].

The observed selective sweep surrounding the GTP cyclohydrolase gene (*pfgch1*; *PF3D7_1224000*) on chromosome 12 is unique to Malawi in this study. The *pfgch1* gene is the first gene in the folate biosynthesis pathway, and adaptive selection could have resulted from SP pressure [40]. A similar phenomenon was previously observed in Thai parasites that evolved reduced microsatellite diversity and increased LD flanking the *pfgch1* locus during SP selection [40]. Further work, such as analysis of copy number variation, may provide better insights into the selection processes at work at this locus, as positive selection may result from rapid spread of chromosomes carrying multiple copies of *pfgch1* [40]. Selection signals in the *trap* gene are thought to reflect genetic adaptation to divergent host ligands [33] involved in the motility of sporozoites and their invasion of hepatocytes and mosquito salivary glands [41].

**Figure 3.**
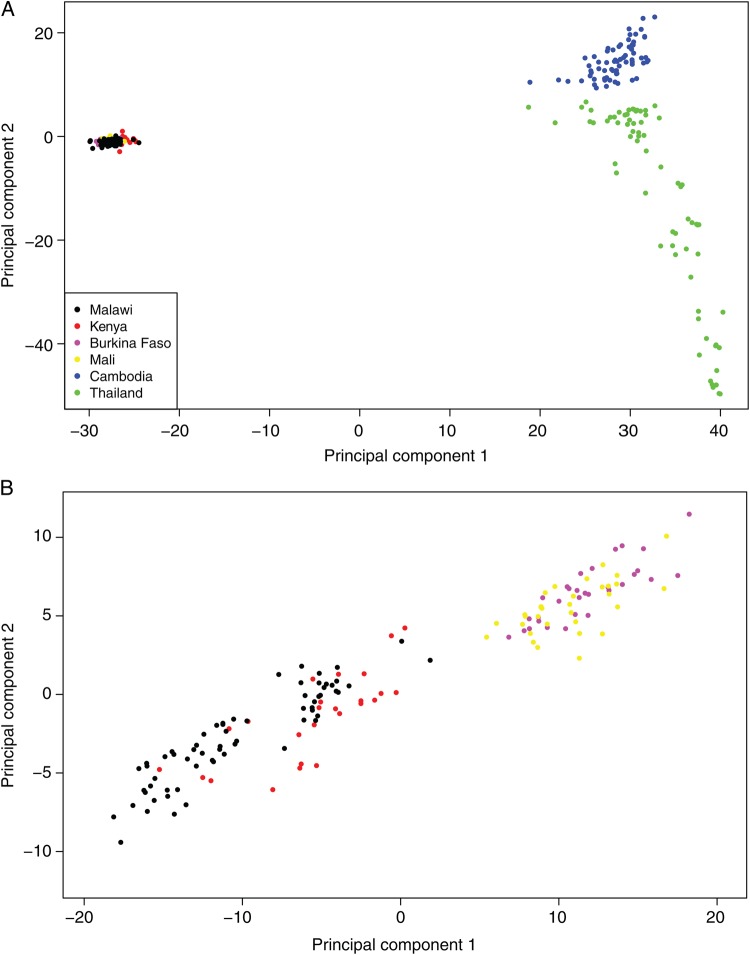
Principal components analysis using single-nucleotide polymorphisms differentiates *Plasmodium falciparum* isolates by continent and within Southeast Asia (*A*) and between East and West Africa (*B*). The proportion of variation explained by the first 2 principal components is 13.4% (*A*) and 3.8% (*B*).

The populations of *P. falciparum* are geographically structured, resulting from adaptation to different environments and selection pressures [16, 18]. Prior to analyzing sequence data for population structure, SNPs in the mitochondrial genome (*mt*; approximately 6 kb) were used to confirm that the Malawian samples were of African origin. Haplotypes were formed using 4 established continent-specific polymorphisms (*mt772, mt1692, mt4179,* and *mt4952*) [42]. Two haplotypes of African origin were present: CGCC (identical to 3D7) and CACC, in 90.8% and 9.2% of samples, respectively. High read-depth coverage in *mt* (median and mean, approximately 1560-fold and 1245-fold, which were approximately 19-fold and 23-fold greater, respectively, than the nuclear genome) was consistent with the known multiple copies of the organelle in a *P. falciparum* cell [43]. There was no obvious clustering of Malawian SNPs (data not shown), probably because all samples were obtained in the same season and district. A PCA of the 294 187 SNPs from all 6 populations revealed expected differences between Africa and Asia, within Southeast Asia, and within Africa (Figure 3), as previously reported [16]. We further applied the SNP-wise *F_ST_* metric to measure genomic divergence across the 6 populations. At a stringent genome-wide cutoff (*F_ST_* ≥ 0.2; top 0.5% overall), we identified the most divergent loci between Malawian samples and the other 5 populations. The frequency of alleles encoding known drug targets and their divergence between populations is shown in Supplementary Table 2 and Table 4.

**Table 4.** Frequency of Alleles Conferring Drug Resistance Across the 6 Populations

Locus, Mutation	Malawi	Kenya	Mali	Burkina Faso	Thailand	Cambodia
CRT						
K76T	0	0.31	0.62	0.36	1	1
Q271E	0	0.23	0.59	0.23	1	0.99
N326S	0	0.05	0.01	0.05	1	0.91
I356T	0	0	0.18	0.09	1	0.92
DHPS						
S436A	0	0.03	0.61	0.49	0.18	0.33
A437G	0	0.18	0.61	0.39	0	0.02
K540E	1	0.81	0	0	0.87	0.41
A581G	0	0	0	0	0.78	0.57
MDR						
N86Y	0.02	0.55	0.26	0.27	0.01	0.02
N1226Y	0	0.01	0	0	0.58	0.01
D1246Y	0	0.43	0.02	0.07	0	0

The interpopulation differences at drug resistance loci likely reflect local historical parasite adaptation to drug pressure, leading to fixation or near fixation of the implicated resistance alleles, as evidenced by the following observations. First, reductions in the prevalence of *pfcrt*-K76T alleles after CQ withdrawal differs between Malawi (0%) and Kenya (31%), a disparity that has been observed previously [44, 45]. The return of CQ-susceptible malaria to Malawi has prompted discussions of the possibility that CQ, once rendered ineffective, may become useful again [46]. Second, the prevalence of *pfcrt*-K76T alleles differs between Mali (62%) and Burkina Faso (36%), where CQ is still used. Third, *pfcrt*-K76T alleles have reached fixation in Cambodia and Thailand; indeed, CQ remains the first-line treatment for *Plasmodium vivax* malaria in these 2 countries and thus may continue to select for the resistant genotype in *P. falciparum* [47]. Fourth, *pfdhps*-K540E alleles have reached fixation in Malawi, despite the drug policy change from SP to ACT in 2007. The persistence of these alleles may be maintained by the use of SP as the first-line drug for IPTp and the use of trimethoprim/sulfamethoxazole, an antifolate antibiotic used widely for various indications. Although *F_ST_* values may reflect differences in allele frequency due to differential selective pressure, they may also simply reflect random genetic drift. Further analysis at the gene level provided evidence of a high correlation between maximum *F_ST_* and XP-EHH (Spearman ρ: overall, 0.181; in Kenya, 0.164; in Burkina Faso, 0.155; in Mali 0.143; in Cambodia, 0.261; and in Thailand, 0.264), suggesting that some signals of directional selection were detected using the population differentiation approach. However, it is possible that extreme XP-EHH could reflect demographic characteristics rather than selection pressure.

Finally, a comparison of our data with those from a recent analysis from West Africa (Gambia and Guinea) highlights several key points about the *P. falciparum* population structure in Africa [48]. First, the predominantly negative Tajima *D* values in the African (Guinean and Malawian) populations indicate a historical population expansion in Africa. Second, balancing selection acts on a similar set of genes (including those predominantly expressed in merozoites and involved in erythrocyte invasion) in Guinean and Malawian samples, suggesting that it acts on similar antigenic targets irrespective of differing population demographic characteristics. For example, *msp3.8* has the highest Tajima *D* value in both Malawi and Guinea. Third, signatures of directional selection at drug resistance loci appear to be population specific as expected, reflecting historical differences in antimalarial drug use. For example, there are strong signatures of directional selection around *pfcrt* in Guinea (where CQ was used until 2006) but not in Malawi (where CQ was withdrawn in 1998). In contrast, there is evidence of strong selection in *pfdhps* in Malawi but only weak selection in Guinea, where SP was never introduced as first-line treatment for malaria [48].

## CONCLUSION

Our study demonstrates how the large and growing number of *P. falciparum* whole-genome sequences can be used to understand malaria biology and impact disease control. Estimates of parasite genetic diversity, LD, and MOI will improve our understanding of various aspects of malaria epidemiology, including infection dynamics, transmission levels, pathogenesis mechanisms, and drug efficacy. For example, the high nucleotide diversity, short-range LD, and MOI found in Malawian samples reflect the high transmission history of the district in which they were collected. Additionally, we have provided insights into the parasite population structure within Malawi and placed it in the context of other populations, facilitating the recognition of potentially imported cases into the country and targeting of effective malaria control measures. Our whole-genome analytical approach has detected evolutionary genetic signatures and genes under selective pressure due to drug resistance or virulence. For example, we observed differences at drug resistance loci between geographically distinct populations due to differing histories of antimalarial drug use. However, we also observed evidence of selection pressure in genes with unknown functions, which can be investigated in future experiments.

## Supplementary Data

Supplementary materials are available at *The Journal of Infectious Diseases* online (http://jid.oxfordjournals.org). Supplementary materials consist of data provided by the author that are published to benefit the reader. The posted materials are not copyedited. The contents of all supplementary data are the sole responsibility of the authors. Questions or messages regarding errors should be addressed to the author.

Supplementary Data
