# Supplementary material for: Whole-Genome Scans Provide Evidence of Adaptive Evolution in Malawian Plasmodium falciparum Isolates
Source: J Infect Dis. 2014 Jun 19;210(12):1991–2000. doi: 10.1093/infdis/jiu349 (PMC4241944; doi:10.1093/infdis/jiu349)
Supplement: Supplementary Data [file supp_210_12_1991__index.html]

Whole-genome scans provide evidence of adaptive evolution in Malawian Plasmodium falciparum isolates — Whole-Genome Scans Provide Evidence of Adaptive Evolution in Malawian Plasmodium falciparum Isolates — Whole-Genome Scans Provide Evidence of Adaptive Evolution in Malawian Plasmodium falciparum Isolates — Supplementary Data 

# Whole-Genome Scans Provide Evidence of Adaptive Evolution in Malawian *Plasmodium falciparum* Isolates

## Supplementary Data

Supplementary Data

**Files in this Data Supplement:**

- Supplementary Data - Docx file
