# Supplementary material for: Whole-Genome Scans Provide Evidence of Adaptive Evolution in Malawian Plasmodium falciparum Isolates
Source: J Infect Dis. 2014 Jun 19;210(12):1991–2000. doi: 10.1093/infdis/jiu349 (PMC4241944; doi:10.1093/infdis/jiu349)

**Supplementary Table 1**

**Regions of at least size 1kb (≥5 SNPs) where linkage disequilibrium is high (average *r^2^*>0.5)**

| Chr | Start | End | Size | Average *r^2^* | Gene (product) |
| --- | --- | --- | --- | --- | --- |
| 2 | 290044 | 291833 | 1789 | 0.539 | PF3D7_0207300 (*SERA8*) |
| 2 | 298059 | 299430 | 1371 | 0.539 | PF3D7_0207500 (*SERA6*) |
| 2 | 610445 | 612321 | 1876 | 0.841 | PF3D7_0214900 (*RON6*) |
| 2 | 848573 | 855644 | 7071 | 0.614 | PF3D7_0221000 (*Plasmodium* exported protein) |
| 4 | 688420 | 689471 | 1051 | 0.638 | PF3D7_0415500 (within 60kb of *pfdhfr*) |
| 4 | 1133341 | 1137027 | 3686 | 0.607 | PF3D7_0425100 (*Plasmodium* exported protein, hyp6) |
| 5 | 62996 | 77831 | 14835 | 0.717 | PF3D7_0501200 (*PIESP2*) |
| 6 | 494710 | 496596 | 1886 | 0.87 | PF3D7_0611800 (Conserved *Plasmodium* protein, UF) |
| 6 | 554922 | 556816 | 1894 | 0.69 | PF3D7_0613600 (Conserved *Plasmodium* protein, UF) |
| 6 | 1114071 | 1116592 | 2521 | 1 | PF3D7_0627800 (*ACS*) |
| 6 | 1235507 | 1238829 | 3322 | 0.895 | PF3D7_0629700 (*SET1*) |
| 7 | 151936 | 153881 | 1945 | 0.692 | PF3D7_0703800:PF3D7_0703900 |
| 7 | 308358 | 309548 | 1190 | 1 | ~100kb from *pfcrt* |
| 7 | 1358524 | 1359785 | 1261 | 0.737 | PF3D7_0731500 (*EBA175*) |
| 8 | 487329 | 489310 | 1981 | 0.82 | PF3D7_0809600 (Peptidase family C50, P) |
| 8 | 530810 | 532060 | 1250 | 0.839 | ~16kb from *pfdhps* |
| 8 | 586054 | 587997 | 1943 | 0.519 | PF3D7_0811600 (Conserved *Plasmodium* protein, UF) |
| 8 | 712889 | 713998 | 1109 | 0.538 | PF3D7_0815100 (RAP protein, P) |
| 8 | 1108383 | 1110060 | 1677 | 0.579 | PF3D7_0826000 (Conserved *Plasmodium* protein, UF) |
| 9 | 430465 | 432073 | 1608 | 0.737 | PF3D7_0909500 (*SPM1*) |
| 9 | 963651 | 964890 | 1239 | 1 | PF3D7_0923800 (Thioredoxin reductase) |
| 9 | 1404908 | 1406161 | 1253 | 0.736 | PF3D7_0935600 (Gametocytogenesis-implicated protein) |
| 10 | 1432739 | 1434129 | 1390 | 0.839 | PF3D7_1036300 (Merozoiote surface protein 3.8) |
| 12 | 407935 | 409617 | 1682 | 0.644 | PF3D7_1208800 (Zinc finger protein, P) |
| 12 | 841250 | 842350 | 1100 | 0.731 | PF3D7_1221000 (Histone-lysine N-methyltransferase |
| 12 | 865159 | 866322 | 1163 | 1 | PF3D7_1221600:PF3D7_1221700 |
| 12 | 940463 | 941821 | 1358 | 0.92 | PF3D7_1223400 (Phospholipid-transporting ATPase, P) |
| 12 | 954490 | 955708 | 1218 | 1 | PF3D7_1223500 (Conserved Plasmodium protein, UF) |
| 12 | 989679 | 991594 | 1915 | 0.917 | PF3D7_1224300 (Polyadenylate-binding protein) |
| 12 | 1084479 | 1085565 | 1086 | 0.783 | PF3D7_1226800 (Ataxin-3, putative) |
| 13 | 445816 | 447295 | 1479 | 1 | PF3D7_1309800 (Conserved Plasmodium protein, UF) |
| 14 | 84654 | 86570 | 1916 | 1 | PF3D7_1402200:PF3D7_1402300 (RPN6) |
| 14 | 756677 | 758451 | 1774 | 1 | PF3D7_1417900 (Conserved Plasmodium protein, UF) |

UF = Unknown function, P = Putative and Chr = Chromosome.

**Supplementary Table 2**

***F_ST_* values at known drug-resistance loci. Blanks infer very low *F_ST_* values.**

| Loci | Mutation | Kenya | Burkina Faso | Mali | Cambodia | Thailand |
| --- | --- | --- | --- | --- | --- | --- |
| *CRT* | K76T | 0.22 | 0.25 | 0.49 | 1 | 1 |
|  | Q271E |  |  | 0.46 | 0.98 | 1 |
|  | N326S |  |  |  | 0.82 | 1 |
|  | I356T |  |  |  | 0.83 | 1 |
|  |  |  |  |  |  |  |
| *DHPS* | S436A |  | 0.35 | 0.47 |  |  |
|  | A437G |  | 0.28 | 0.49 |  |  |
|  | K540E |  | 0.91 | 0.91 | 0.34 |  |
|  | A581G |  |  |  | 0.37 | 0.61 |
|  |  |  |  |  |  |  |
| *MDR1* | N86Y | 0.37 |  |  |  |  |
|  | N1226Y |  |  |  |  | 0.37 |
|  | D1246Y | 0.32 |  |  |  |  |

**Supplementary Figure 1**

**Minor allele frequency spectrum of different classes of nucleotide sites showing excess of rare alleles, and indicating potential population growth in Malawi in the recent past. Neutral refers to a Wright Fisher model of constant population size**


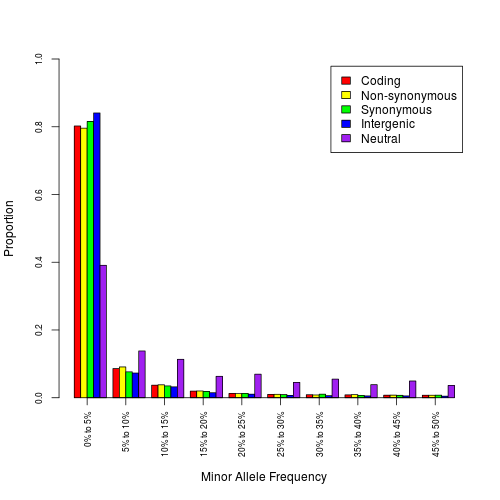

Supplement: Supplementary Data [file supp_jiu349_jiu349supp.docx]
